# *In vivo* behavior of [^64^Cu]NOTA-terpyridine platinum, a novel chemo-radio-theranostic agent for imaging, and therapy of colorectal cancer

**DOI:** 10.3389/fmed.2022.975213

**Published:** 2022-09-23

**Authors:** Meysam Khosravifarsani, Samia Ait-Mohand, Benoit Paquette, Léon Sanche, Brigitte Guérin

**Affiliations:** ^1^Department of Nuclear Medicine and Radiobiology, Faculty of Medicine and Health Sciences, Université de Sherbrooke, Sherbrooke, QC, Canada; ^2^Sherbrooke Molecular Imaging Center (CIMS), Centre de Recherche du CHUS (CRCHUS), Université de Sherbrooke, Sherbrooke, QC, Canada

**Keywords:** terpyridine platinum complexes, copper-64, radio-theranostic agent, resistance, colorectal cancer

## Abstract

To overcome resistance to chemotherapy for colorectal cancer, we propose to validate *in vivo* a novel terpyridine-platinum (TP) compound radiolabeled with the radio-theranostic isotope ^64^Cu. *In vivo* stability, biodistribution, PET imaging, tumor growth delay, toxicity and dosimetry of [^64^Cu]NOTA-C3-TP were determined. The current experimental studies show that [^64^Cu]NOTA-C3-TP is stable *in vivo*, rapidly eliminated by the kidneys and has a promising tumor uptake ranging from 1.8 ± 0.4 to 3.0 ± 0.2 %ID/g over 48 h. [^64^Cu]NOTA-C3-TP retarded tumor growth by up to 6 ± 2.0 days and improved survival relative to vehicle and non-radioactive [^Nat^Cu]NOTA-C3-TP over 17 days of tumor growth observation. This effect was obtained with only 0.4 nmol *i.v*. injection of [^64^Cu]NOTA-C3-TP, which delivers 3.4 ± 0.3 Gy tumoral absorbed dose. No evidence of toxicity, by weight loss or mortality was revealed. These findings confirm the high potential of [^64^Cu]NOTA-TP as a novel radio-theranostic agent.

## Graphical Abstract

Long tumor retention time and tumor growth inhibition from a novel chemo-radio-theranostic agent. 
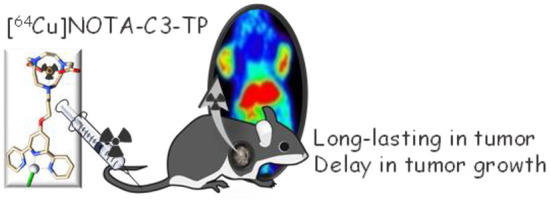

## Introduction

Despite large-scale screening, colorectal cancer (CRC) is still the third cause of cancer related death and fourth most diagnosed cancer (1, 2). CRC emerges from epithelial and glandular cells, which carry genetic or epigenetic mutations (3). Early stage (I-II) cancer patients are mostly cured with surgery as the principal modality of treatment. However, in high stage (IV) patients, when the cancer cells are spread throughout the body, systemic chemotherapy and radiation therapy are administered in combination with surgery (4). Despite great advances with chemotherapeutic agents for systemic treatment, the 5-year survival rate of patients is still only 10% for high-stage CRC (5). 5-fluorouracil (5-Fu) has been suggested as the major chemotherapeutic agent for CRC treatment. In mismatch repair deficient patients who are resistant to 5-Fu, oxaliplatin alone or in combination with 5-Fu has been suggested as the main therapeutic agents (6). The main reason for poor response to chemotherapy treatment is associated to an intrinsic and acquired resistances to chemotherapeutic agents, which occur in ~90% of the CRC patients (7). Several processes can lead to the development of resistance to platinum-based drugs (7). These include a decrease in cellular uptake of drug, an increased in efflux activity, inactivation of the platinum agent by thiol containing small peptides including glutathione (GSH) and metallothionein (MT), enhanced nucleotide excision repair (NER), and increased expression of anti-apoptotic proteins such as P53 (p21/waf1) and bcl-2 (8). To overcome these resistances and reduce toxicity associated with most platinum compounds (e.g., cisplatin and oxaliplatin), a myriad of platinum-based drugs has been developed with different structure and mechanism of action (9, 10).

Among these drugs, the G-quadruplex (G4) DNA binders, such as square planar agents like terpyridine platinum (TP)-based compounds, have attracted considerable recent attention (11). Given the specific structure of G4 and its presence in telomers and on the human promotor of oncogenes, many consider that G4 binders could act as new therapeutic targets (12). The TP compounds interact with DNA quadruplexes mainly *via* π-π stacking with the exterior G-tetrad and replacement of monovalent alkaline ions found in the central channel of G-quadruplexes (13). For instance, a bimetallic TP-based introduced by Stafford et al. exhibited up to 1,000-fold higher affinity toward G-quadruplex relative to other intermixed sequence of DNA (14).

In recent studies, we have presented the first proof-of-concept for a novel class of chemo-radio-theranostic agents composed of [^64^Cu]NOTA-TP conjugates on human CRC cells (15, 16). Following these studies, we note that a paper reported the characterization a platinum based G4 DNA binder radiolabeled with ^111^In for SPECT imaging (17).

**Figure 1:**
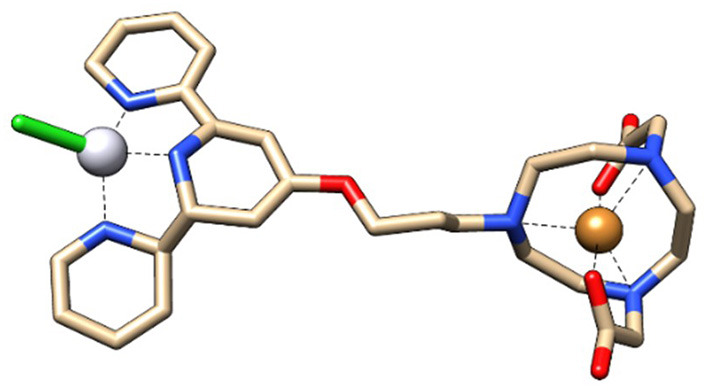
3D Structure of [^64^Cu]Cu-C3-NOTA-TP conjugate (Chimera software 1.15).

The aim of combining the ^64^Cu, a strong radio-theranostic agent [*T*_1/2_ = 12.7 h; EC, (43.1%), β^+^, 0.653 MeV (17.8%); β^−^, 0.579 MeV (38.4%)], with NOTA-TP in a single molecule (15, 16) was to optimize the concomitant effect of Pt drug and radiotherapy for potential use in clinic (18). The lesions induced by highly destructive secondary low energy electrons (LEEs) generated from ^64^Cu and DNA modifications due to intercalation of the Pt drug NOTA-TP into G-quadruplex (12) should maximize the DNA damages. Accordingly, the cytotoxicity of [^64^Cu]NOTA-TP conjugates at high apparent molar activity, was 27,800- to 55,000-fold greater than that of [^Nat^Cu]NOTA-TP in the HCT116 colorectal cancer cell line, supporting a strong effect by the combination in the same molecule of ^64^Cu and the TP residue (15, 16). These solid *in vitro* proof-of-principle studies (15, 16) support examination of [^64^Cu]NOTA-TP in strategies to develop innovative PET imaging and therapeutic tools for the management of CRC. Additionally, given that a higher cytotoxic activity and selectivity toward HCT116 cells were previously obtained with the flexible linker derivative of [^64^Cu]NOTA-C3-TP, this latter was selected for the present *in vivo* study (Figure 1).

The interaction of LEEs originated from Auger electron-emitting ^64^Cu with the monofunctional adducts formed between NOTA-TP and DNA could generate reactive radical species that mostly interact with the nearby DNA subunits, particularly guanine and adenines (19). This phenomenon will form intra- and inter-strand cross links on vulnerable domains of DNA (i.e., G-quadruplexes) that are challenging for the DNA damage repair system and can induce cell apoptosis (20). Rezaee et al. demonstrated that a single hit by a LEE (E~0.5eV) could generate double strand breaks (DSBs) in cisplatin-modified DNA, but not in pure DNA (21).

The current study investigates the *first-in-animal* chemoradio-theranostic (CRT) effect of [^64^Cu]NOTA-C3-TP. Balb/c mice were used to assess the *in vivo* stability and the tissue biodistribution from 4 to 48 h post-injection. The tumor uptake and tissue distribution were also determined by whole-body positron emission tomography (PET) imaging in on a nude (nu/nu) mouse model implanted with the human CRC cells (HCT116). Additionally, tumor growth delay, toxicity and dosimetry of [^64^Cu]NOTA-C3-TP was determined in mice carrying the HCT116 tumor. Finally, a comparison of the selectivity and antitumor efficiency of [^64^Cu]NOTA-C3-TP vs. that of a combination treatment of oxaliplatin and external beam radiation therapy was made for CRC of HCT116 tumor.

## Results

### Chemistry and radiolabeling

**Figure 2:**
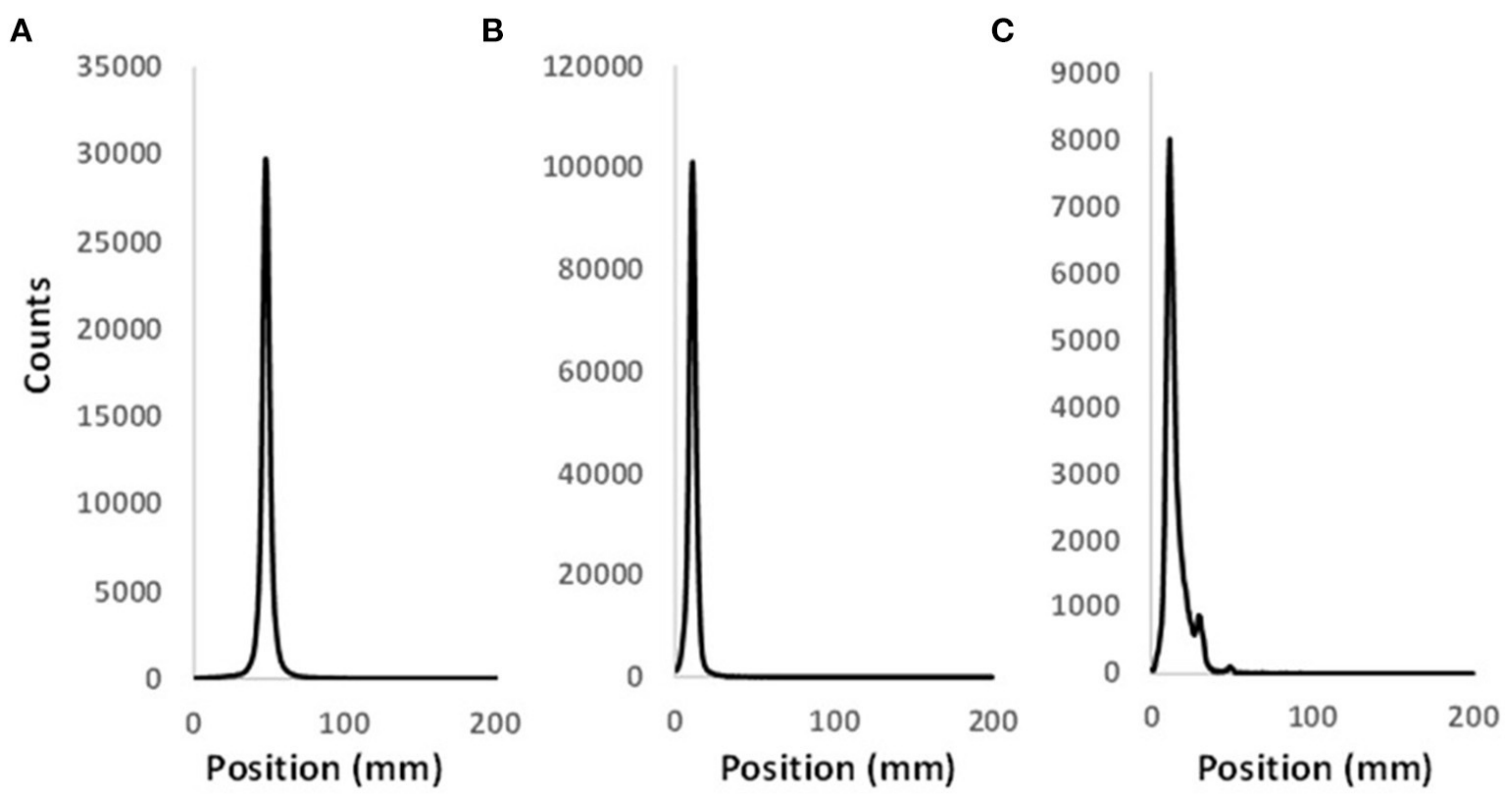
Representative radio-TLC profiles of pure **(A)** [^64^Cu](OAc)_2_ and **(B)** [^64^Cu]NOTA-C3-TP. **(C)** Radio-TLC profile of [^64^Cu]NOTA-C3-TP in the whole plasma 48 h after its injection in Balb/c mouse.

Synthesis of NOTA-C3-TP, [^Nat^Cu]NOTA-C3-TP and [^64^Cu]NOTA-C3-TP were previously described by our group (16). NOTA-C3-TP and [^Nat^Cu]NOTA-C3-TP are >95% pure by UPLC analysis (Supplementary Figures S1A,B). [^64^Cu]NOTA-TP was obtained in high radiochemical yield (Figure 2B) and AMA (~70–150 MBq/nmol).

### *In vivo* stability of [^64^Cu]NOTA-TP

As control, the [^64^Cu]Cu(OAc)_2_ and [^64^Cu]Cu-NOTA-C3-TP were analyzed by radio-TLC to determine their respective retention times (Figures 2A,B). The radio-TLC profiles of the whole plasma indicate that 1% of ^64^Cu was released 48 h after injection (Figure 2C). A new peak with an intensity of 9% appearing could be related to the conjugation of the radio-theranostic agent with plasma proteins or formation of a metabolite of [^64^Cu]NOTA-C3-TP (Figure 2C). To explain these results, [^64^Cu]NOTA-C3-TP was incubated *ex vivo* in plasma during 48 h and then analyzed by radio-TLC with and without protein precipitation (Supplementary Figure S2). Two peaks were observed on the radio-TLC of the whole plasma (Supplementary Figure S2A). [^64^Cu]NOTA-C3-TP and trace amount of free ^64^Cu corresponding to 4% of the total ^64^Cu activity were observed on radio-TLC of plasma after protein precipitation (Supplementary Figure S2B). These results support the progressive binding of [^64^Cu]NOTA-C3-TP to plasma proteins *in vivo*.

It is noteworthy to mention that the amount of [^64^Cu]NOTA-C3-TP bound to plasma protein (*ex vivo*) reached 96% at 24 h (Supplementary Table S2), which was similar than measured with standard platinum compounds such as cisplatin (98%) and oxaliplatin (98%) at the same time (22, 23).

The ability of [^64^Cu]NOTA-C3-TP to bind to plasma proteins was also assessed by comparing the absorbance spectra of [^64^Cu]NOTA-C3-TP and those of collected whole plasma alone or in presence of the radio-theranostic agent (Supplementary Figure S3). The binding of [^64^Cu]NOTA-C3-TP to plasma proteins has resulted in a significant increase at wavelengths between 310 and 380 nm (Supplementary Figure S3B, green line). For instance, the absorption intensity for pure plasma has increased from 0.09 (Supplementary Figure S3A, green line) to 1.56 when assessed with plasma proteins of 50–70 KD (Supplementary Figure S3B, green line) at 333 nm, which corresponded to an absorbance peak of [^64^Cu]NOTA-C3-TP (Supplementary Figure S3A, red line). Since the absorption intensity was similar to those measured with pure [^64^Cu]NOTA-C3-TP at same concentration (4 mM), this suggested that the majority of [^64^Cu]NOTA-C3-TP complex was bound to the 50–70 KD plasma proteins.

### Biodistribution and pharmacokinetic studies in Balb/c mice

**Figure 3:**
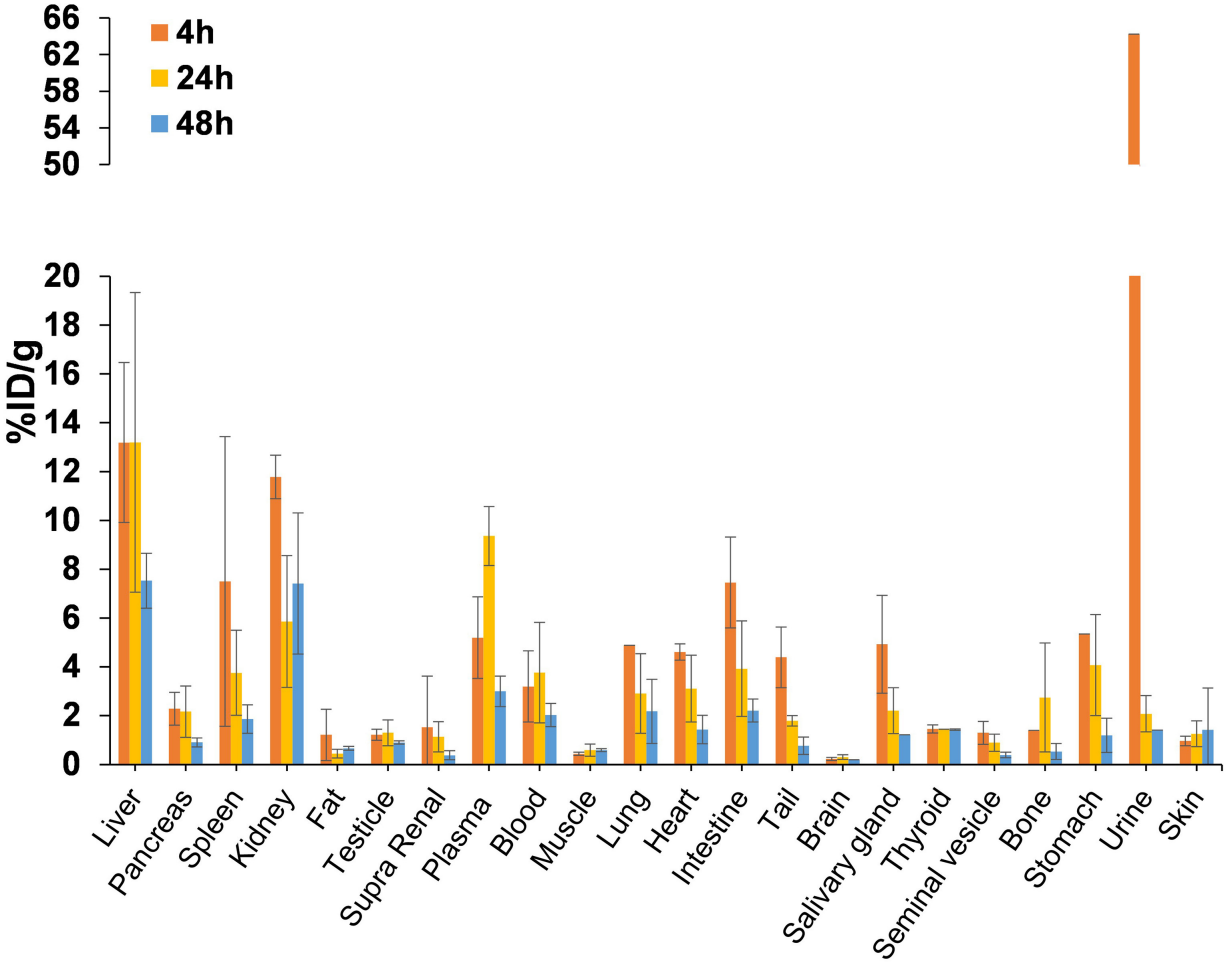
Biodistribution profile of [^64^Cu]NOTA-C3-TP in Balb/c mice (*n* = 3/group) at 4, 24, and 48 h *p.i*.

Biodistribution studies were carried out in Balb/c mice at 4, 24, and 48 h post-injection (*p.i*.) to estimate the pharmacokinetic profile of the tracer and best time for PET imaging (Figure 3). [^64^Cu]NOTA-C3-TP was rapid eliminated *via* kidney into the urine, as shown by a drastic reduction from 64.22 ± 5.89 percentage of injected dose per gram (%ID/g) at 4 h *p.i*. to only 2.06 ± 1.91 %ID/g at 24 h, followed by a further decrease to 1.4 ± 0.6 %ID/g at 48 h. The highest uptakes were 13 ± 3 and 11.7 ± 0.9 %ID/g in the liver and kidney at 4 h *p.i*., which decreased to 7.5 ± 2.1 and 7.4 ± 5.3 %ID/g at 48 h, respectively. Interestingly, the corresponding values for the other organs remained below 3% at 48 h, which indicates a low retention of the [^64^Cu]NOTA-C3-TP. The [^64^Cu]NOTA-C3-TP measured in plasma was 5.2 ± 2.0 %ID/g at 4 h, and then significantly increased to 9.4 ± 2.1 at 24 h (*p* < *0.05*) followed by a reduction to 3.0 ± 1.1 %ID/g at 48 h (*p* < *0.01*; Figure 3).

### PET imaging in HCT116 tumor-bearing nude mice

**Figure 4:**
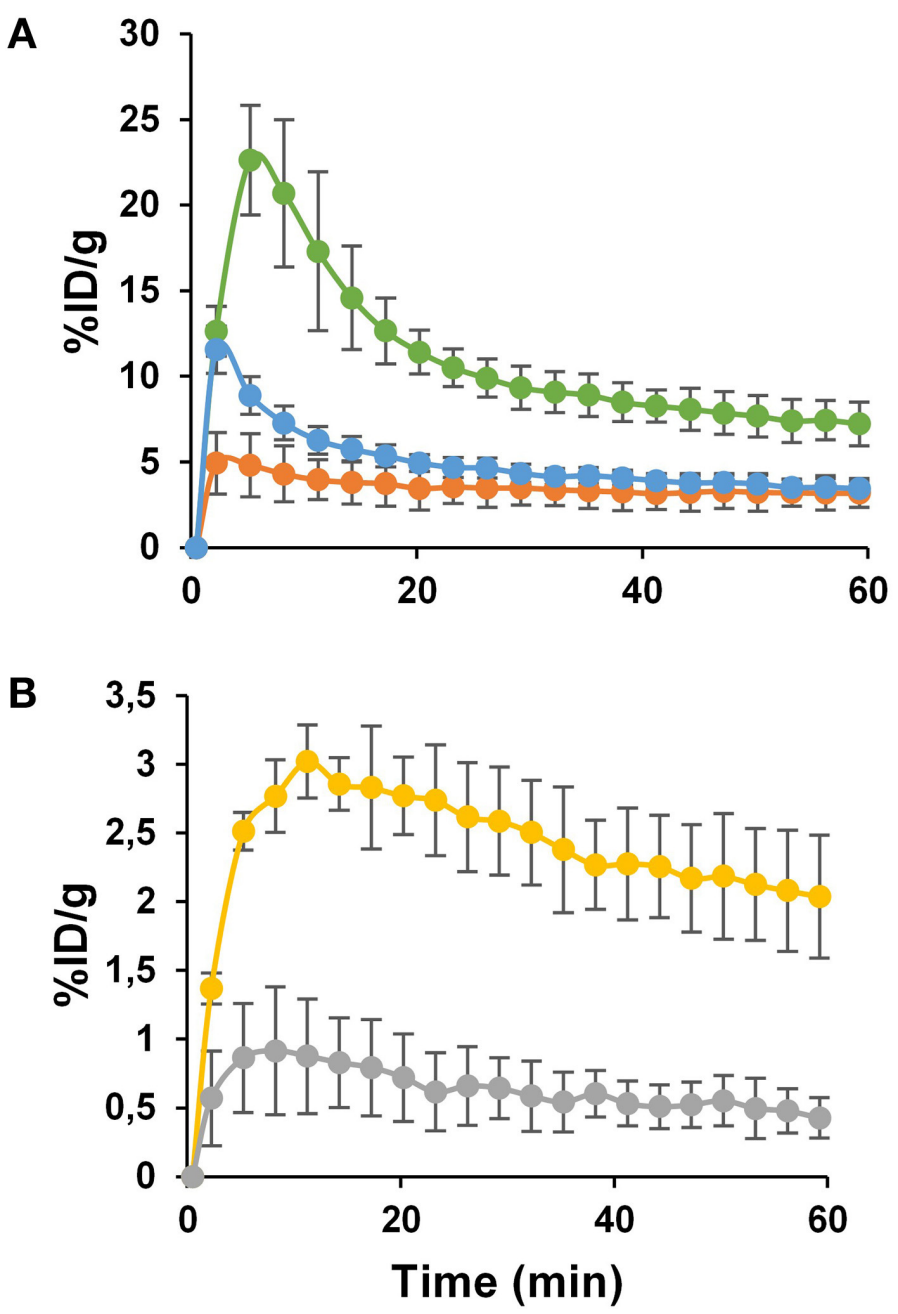
The kinetics of PET image-derived uptakes (%ID/g) ± SD in kidney (**A**, green line), liver (**A**, orange line), blood (**A**, blue line), tumor (**B**, yellow line), and muscle (**B**, gray line) measured during 1 h dynamic scan. Each data point represents the average values for 7 nude mice.

Accumulation of [^64^Cu]NOTA-C3-TP in HCT116 tumor implanted in nude mice was assessed with a PET imaging scanner. A dynamic scan was performed during the 1st h after the injection to estimate the accumulation of [^64^Cu]NOTA-C3-TP in tissues (%ID/g) after conversion of the regions of interest (ROIs) traced around liver, kidney, muscle, and the tumor (Figure 4). During the 1st h after injection, tumor uptake and retention ranging from 2.0 ± 0.4 to 3.0 ± 0.2 %ID/g (Figure 4B, yellow line) were associated with a very fast elimination from the kidney (Figure 4A, green line).

Indeed, the quantity of [^64^Cu]NOTA-C3-TP in kidney rapidly decreased from 22 ± 5 %ID/g at 5 min post-injection to 7.2 ± 1.1 %ID/g at 1 h. The radio-theranostic agent was slowly eliminated from the liver compared to kidney, since the %ID/g diminished from 4.9 ± 1.6 at 2 min to 3.1 ± 0.5 at 1 h *p.i*. (Figure 4A, orange line). This result indicates 2.3 to 4.4-fold higher kidney uptake relative to liver during the 1-h dynamic scan. The %ID/g in blood followed a fast elimination kinetic pattern with an initial peak of 11 ± 2 %ID/g at 2 min to 3.4 ± 1.0 %ID/g at 1 h (Figure 4A, blue line). In the muscle, the %ID/g has remained below 1% ranging from 0.9 ± 0.3 to 0.4 ± 0.1 %ID/g during the whole dynamic scan (Figure 4B, gray line).

**Figure 5:**
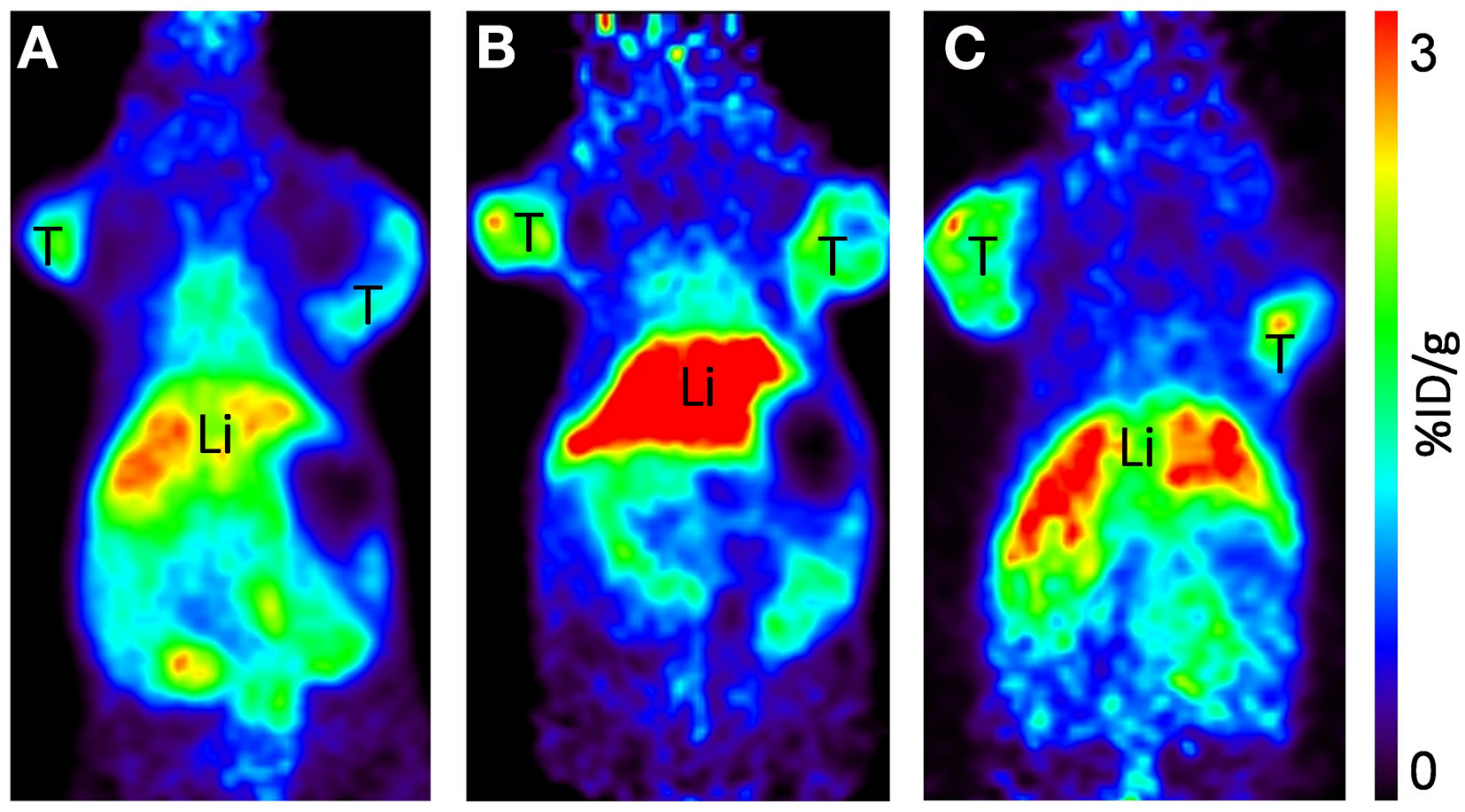
Representative coronal PET images of [^64^Cu]-NOTA-C3-TP injected at 30 MBq, at 4 **(A)**, 24 **(B)**, and 48 h **(C)** *p.i*. in HCT116 human colorectal cancer bearing mice. T, tumor; Li, liver. All images are decay-corrected and adjusted to the same maximum value.

Static scans were done at 4, 24, and 48 h *p.i*. of [^64^Cu]NOTA-C3-TP (Figure 5; Supplementary Figure S4). Comparison of all coronal images clearly shows that small percentages of [^64^Cu]NOTA-C3-TP remained in healthy organs at 4, 24, and 48 h *p.i*. Accordingly, the maximum accumulation of [^64^Cu]NOTA-C3-TP was observed in the liver and kidneys, which was < 3 %ID/g. Accumulation in the tumor was comparable to that in critical organs of elimination at all times.

**Figure 6:**
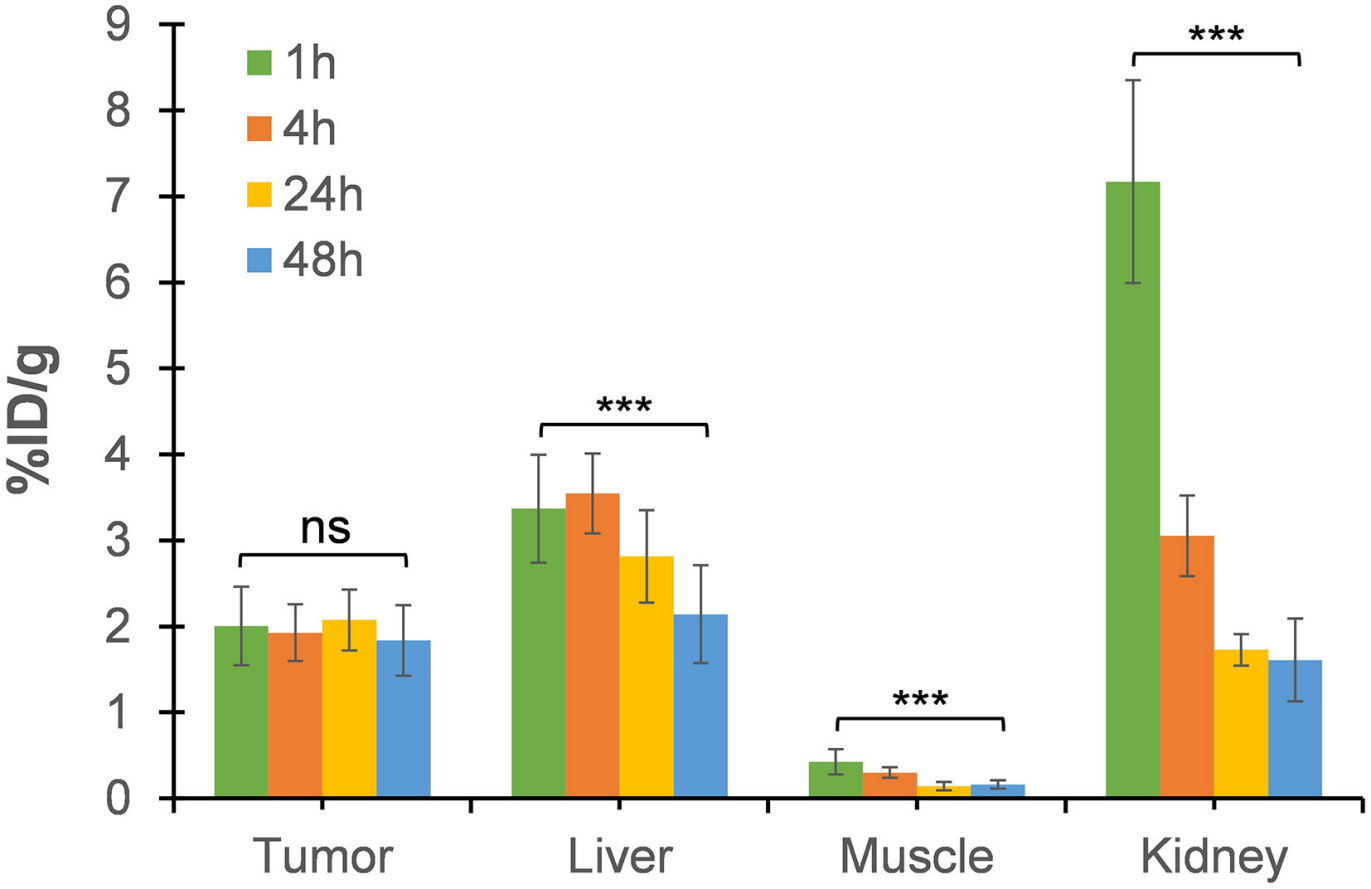
The uptake value (%ID/g) in tumor, liver, muscle, and kidney derived from static PET images at 4, 24, and 48 h post-injection relative to that of 1-h dynamic scan. Each data point represents the averages ± SD for 7 HCT116 tumor-bearing nude mice (***p value < 0.001; ns, not significant).

The tumor uptake percentages (%ID/g) at 4, 24, and 48 h post-injection, which were, respectively, 1.92 ± 0.32, 2.07 ± 0.35, and 1.83 ± 0.40, did not show any significant reduction relative to that at 1-h (*p* > *0.05*; Figure 6). This retention of [^64^Cu]NOTA-C3-TP in the tumor during the first 48 h after injection suggests that ^64^Cu with a physical half-life of 12.7 h would have deposited most of its energy during this period. On the contrary, a rapid elimination from muscle was measured (Figure 6). Elimination of the radio-theranostic agent in the most critical organs kidney and liver was also significant (*p* < *0.001* for both organs) at 48 h post-injection relative to that for 1 h scan. The kidney was the major organ for drug elimination up to 1 h, but the pathway has shifted toward the liver at 4 h with a significantly higher uptake value (*p* < 0.001). The liver uptake can be explained by gradual interaction of [^64^Cu]NOTA-C3-TP with plasmatic proteins, specially serum albumin, that increased the molecular weight and change charge of the complex (24). *In vivo* PET-derived tumor to liver ratios were very stable over time (0.63 ± 0.13 at 1 h vs. 0.91 ± 0.33 at 48 h), while the tumor to muscle and the tumor to kidney ratios have increased by three times and seven times during the same period, respectively (Supplementary Table S1).

**Figure 7:**
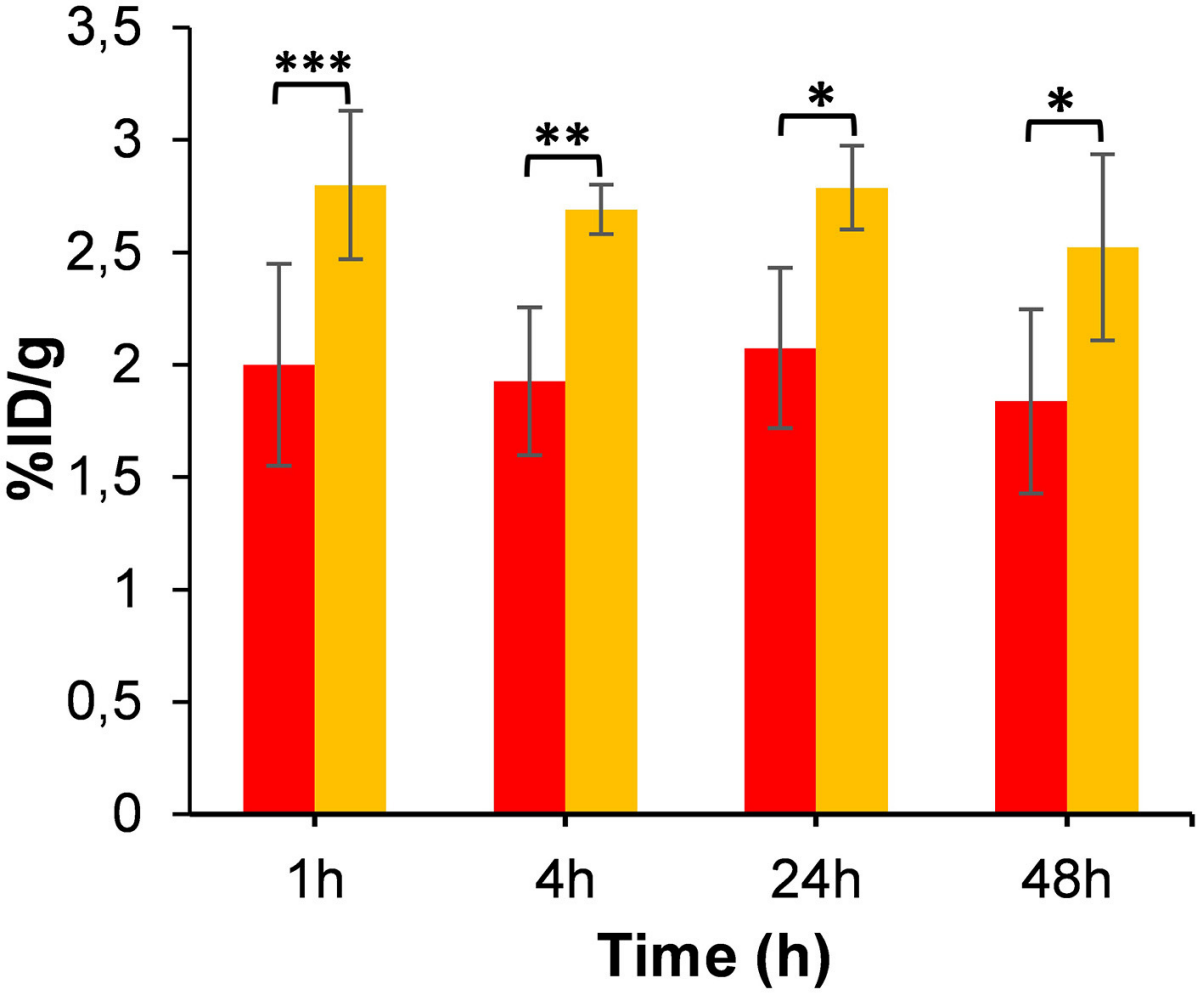
Tumor uptake of [^64^Cu]NOTA-C3-TP (%ID/g) derived from static PET images at 4, 24, and 48 h post-injection relative to that of 1-h dynamic scan (*n* = 4). Red and yellow bars represent tumor uptake without and with co-injection of [^Nat^Cu]NOTA-C3-TP (100-fold excess), respectively (* p value < 0.05, ** p value < 0.01, *** p value < 0.001).

As a test of binding specificity, we imaged animals after co-injection of [^64^Cu]NOTA-C3-TP with a 100-fold excess amount of [^Nat^Cu]NOTA-TP (~40 nmol). Surprisingly, the tumor accumulation was significantly enhanced (~1.4-fold) at all time points post-administration (Figure 7).

The selectivity toward tumor (or tumor-to-organ ratio calculation) of [^64^Cu]NOTA-TP relative to oxaliplatin (18) was also evaluated to estimate the potential therapeutic efficacy of the new complex (Table 1). Using the same HCT116 mouse model, evaluation of [^64^Cu]NOTA-TP in tumor, liver and kidney were similar as the ratios tumor/liver and tumor/kidney at 24 and 48 h post-injection were close to 1, which was between 3.5 and 6.3 times better than oxaliplatin (Table 1). A 12.3–15.2-time higher tumor accumulation relative to muscle was obtained for [^64^Cu]NOTA-TP, which indicate 9–10-fold higher selectivity relative to oxaliplatin (Table 1, entries 1 and 2).

**Table 1:** Selectivity index (or therapeutic window) of [^64^Cu]NOTA-TP relative to standard platinum compounds in clinic.

**Entry**	**Compound**	**Tumor-to-kidney**		**Tumor-to-liver**		**Tumor-to-muscle**	
		**24 h**	**48 h**	**24 h**	**48 h**	**24 h**	**48 h**
1	[^64^Cu]NOTA-TP	1.2	1.13	0.74	0.85	15.2	12.3
2	Oxaliplatin^(18)^	0.25	0.22	0.3	0.22	1.46	1.3

To validate PET results, biodistribution of [^64^Cu]NOTA-C3-TP was performed at 48 h *p.i*. in nude mice implanted with the HCT16 tumor (Supplementary Figure S5). The tumor uptake measured from the biodistribution was 1.54 ± 0.40 %ID/g, which is similar to what was measured by PET imaging (1.83 ± 0.40 %ID/g) at 48 h *p.i*. This correlation between these two methods to quantify the uptake of [^64^Cu]NOTA-C3-TP was also observed for the liver and kidney (Figure 5; Supplementary Figure S5). For the other organs, except plasma, stomach and lung, the uptake was below 1 %ID/g. It is noteworthy that the plasma uptake of [^64^Cu]NOTA-C3-TP at 48 h *p.i*. was only 1.56 ± 0.30 %ID/g in nude mice implanted with the HCT116 tumor, which was 1.9-fold lower than measured in tumor-free Balb/c mice (3.0 ± 1.1 %ID/g, Figure 3; Supplementary Figure S5). Plasma uptake value in HCT116 tumor-bearing nude mice at 48 h was 1.5 %ID/g (Supplementary Figure S5) which was lower than the obtained for tumor-free Balb/c mice (3.0 ± 1.1 %ID/g) at the same time point. This can be explained by about 2–3 %ID/g tumor uptake and retention of complex in the tumor which can reduce uptake value in plasma (Figure 6).

### Radiation dosimetry measurements

The absorbed dose in liver, kidney and tumor were calculated with the OLINDA software (2.1) (25). The estimated absorbed dose to liver and kidney in a 25 g mouse model were, respectively, 35.8 ± 1.0 and 43.4 ± 3.2 milligray/megabecquerel (mGy/MBq), while the total body absorbed dose was 5.1 ± 0.2 mGy/MBq (Table 2). The absorbed dose of tumor, for a selected sphere model with mass of ~0.4 g, was 24.3 ± 4.2 mGy/MBq (Table 2). Accordingly, for injected activities of 70 MBq (1.89 mCi) and 137 MBq (3.7 mCi), the final estimated absorbed doses delivered during this study to the tumor were 1.7 ± 0.3 and 3.4 ± 0.3 Gy. These radiation doses to the tumor were similar to those calculated for kidney and liver, which were, respectively, 3.0 ± 0.2 and 2.56 ± 0.06 Gy and two times higher for 137 MBq. The calculated doses to the tumor were approximately similar to one fraction of radiation delivered during long term (2 Gy) and short term chemoradiotherapy (4 Gy) of colorectal cancer with an external radiation beam in clinic (26).

**Table 2:** Comparison of estimate absorbed dose of nude mice and human from [^64^Cu]NOTA-TP based on nude mouse biodistribution and PET-derived uptake data (*n* = 4).

**Target organs**	**Nude mouse absorbed dose (mGy/MBq)**	Absorbed dose for human (mGy/MBq)*	Effective dose for human (mSv/MBq)*
Brain	3.23	6.84 × 10^−04^	6.84 × 10^−06^
Small intestine	5.6	9.80 × 10^−03^	9.04 × 10^−05^
Stomach wall	4.21	2.07 × 10^−04^	2.48 × 10^−05^
Heart	7.12	4.44 × 10^−04^	4.10 × 10^−06^
Kidney	43.4	6.55 × 10^−03^	5.90 × 10^−05^
Liver	35.8	3.26 × 10^−03^	1.30 × 10^−04^
Lung	18.40	9.18 × 10^−04^	1.10 × 10^−04^
Pancreas	2.12	4.61 × 10^−04^	4.26 × 10^−06^
Skeleton	0.72	1.31 × 10^−04^	1.57 × 10^−05^
Spleen	0.59	2.05 × 10^−04^	1.89 × 10^−06^
Testis	0.51	5.17 × 10^−05^	2.07 × 10^−06^
Thyroid	0.10	5.35 × 10^−05^	2.14 × 10^−06^
Tumor	24.3	-	-
Remainder	5.16	6.91 × 10^−04^	-

^*^Estimated values by OLINDA software (ICRP 103).

Standard deviations are < 5% of the average values.

The extrapolated area under the curve (AUC) value for a human weighting 73 Kg was then calculated to estimate the absorbed doses. The calculated effective doses in liver and kidney were 1.3 × 10^−4^ ± 0.1 × 10^−4^ and 5.9 × 10^−5^ ± 4 × 10^−6^ mSv/MBq, respectively, and the total body effective dose reached 7.54 × 10^−4^ ± 0.3 × 10^−4^ mSv/MBq (Table 2).

### Tumor response after chemo-radiation therapy

**Figure 8:**
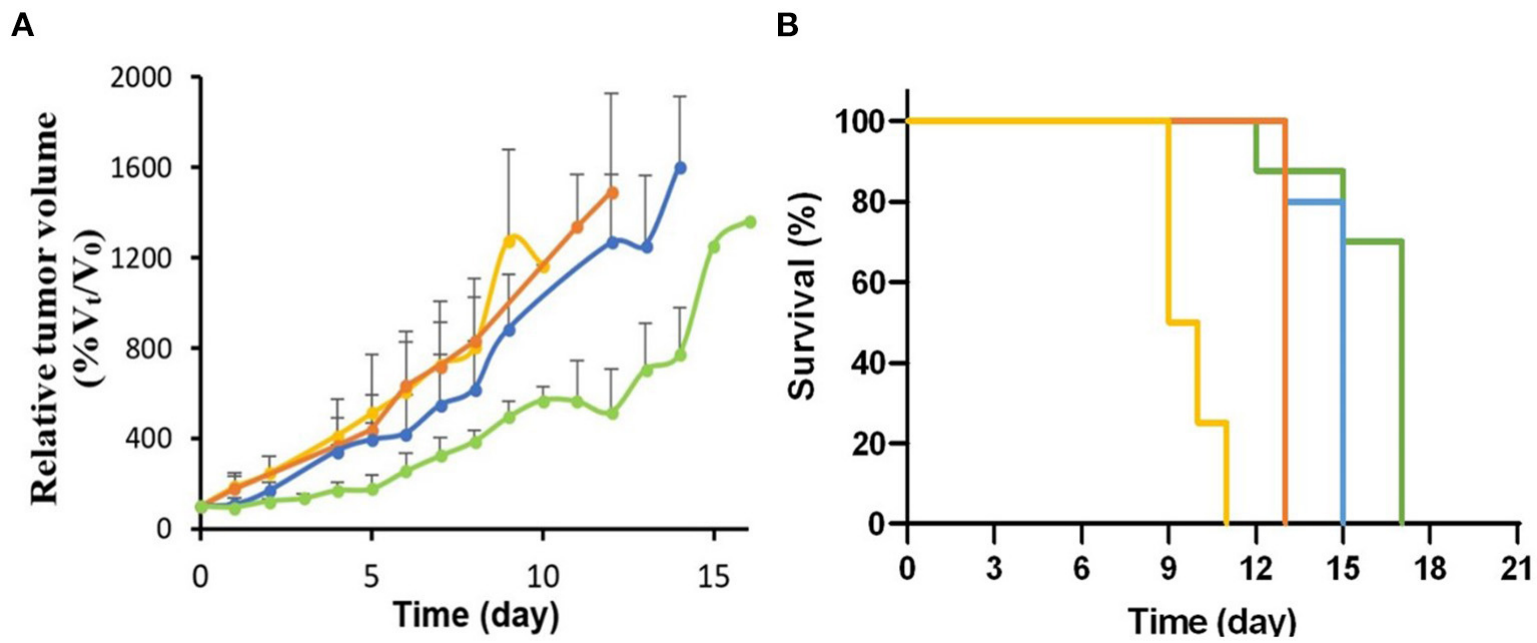
**(A)** Relative tumor growth (%V_t_/V_0_) after treatment with 70 MBq (*n* = 4, blue line) and 137 MBq (*n* = 5, green line) of [^64^Cu]NOTA-C3-TP compared to [^Nat^Cu]NOTA-C3-TP (*p* < 0.001, *n* = 5, orange line) and the vehicle (*p* < 0.001, *n* = 5, yellow line). **(B)** Survival curves between these groups.

The CRT efficacy was determined by measuring the tumor growth after a single *i.v*. injection of 70 or 137 MBq (~0.4 nmol) of [^64^Cu]NOTA-C3-TP in HCT116 tumor bearing nude mice (*n* = 5). These CRT groups were compared to control groups that received either the vehicle (ammonium acetate buffer) (*n* = 5) or [^Nat^Cu]NOTA-C3-TP (*n* = 5). As expected, there was no significant difference between these two control groups (Figure 8A). A significant reduction in relative tumor growth (%V_t_/V_0_), where V_t_ and V_0_ represents tumor volume at time t and 0 correspondingly, in the CRT group treated with 70 MBq compared to control groups was measured on day 1 after drug injection. The relative tumor volumes were, respectively, reduced from 186 ± 63 to 176 ± 57 for the vehicle and [^Nat^Cu]NOTA-C3-TP groups to 107 ± 31 in the 70 MBq CRT group (*p* < 0.05). For the other time points, a reduction in the relative tumor volume was observed for this CRT group relative to the control groups but the difference was not statistically significant (*p* > 0.05).

A significant improvement in the antitumor activity for all time points [days 3–14 (*p*<*0.05*)] was obtained when the injected activity of [^64^Cu]NOTA-C3-TP was escalated to 137 MBq. In addition, the tumor volume did not significantly increase during the first 3 days post-injection (*p* > *0.05*). Kaplan-Meier survival curve indicated a significant difference between the groups (*p* < 0.0001, Figure 8B).

The time required for the tumor volume to increase by 5-fold relative to the initial volume (5Td) was 4.7 ± 0.5 and 5.2 ± 0.5 days for the vehicle and [^Nat^Cu]NOTA-C3-TP groups, which significantly increased to 6.6 ± 1 and 9 ± 0.5 days for the CRT groups injected with 70 MBq or 137 MBq of [^64^Cu]NOTA-C3-TP, respectively. From these 5Td values, the calculated growth delays (TGD) are, respectively, 3.8 ± 0.5 and 4.3 ± 1.0 days for the CRT group that received 137 MBq compared to the vehicle and [^Nat^Cu]NOTA-C3-TP groups.

On day 5 after the injection, tumor volume for the group treated with 137 MBq [^64^Cu]NOTA-C3-TP began to grow at a rate close to that of the mice treated with the non-radioactive [^Nat^Cu]NOTA-C3-TP. Considering a half-life of 12.7 h for ^64^Cu, on day 5 only about 0.2 MBq remained of the 137 MBq that was initially injected. By converting the initial activity into an equivalent dose rate delivered to cells at the perimeter of the primary tumor (assuming a diameter of 3–4 mm), the dose rate delivered on day 1 would be 6.3 mSv/min. Due to the relatively short half-life of ^64^Cu, the dose rate on day 5 would be reduced to 9.1 μSv/min, which represents a 1,000-fold decrease from the initial value (27). This dose rate is also six orders of magnitude lower than that used in clinical radiotherapy (28). Thus, by day 5, the quantity of ^64^Cu remaining in the tumor and the associated dose rate, are insufficient to induce any anti-tumoral effect, suggesting that to limit further tumor growth, a 2nd dose of [^64^Cu]NOTA-C3-TP should be injected by day 5. Note that the same pattern of reduction will occur in the case of average dose rate across the whole tumor mass.

There was no evidence of weight loss (Supplementary Figure S6) and mortality in the CRT groups over the 16 days of tumor growth survey.

## Discussion

Over the years attempts have been made to reduce the toxicity associated with the first generation of platinum-based chemotherapeutic agents (i.e., cisplatin) (7). Newer drugs, including oxaliplatin and carboplatin do show similar therapeutic effect with less toxicity but neurotoxicity and myelosuppression remain major issues for cancer patients treated with even these platinum derivatives (7). Recently, G-quadruplex (G4) bimetallic binders, that exhibit promising cytotoxicity and selectivity toward cancer cells, have emerged (11). Copper and platinum conjugated with planar molecules, are among the most common G4-binders (29). Examples of a radioactive metal conjugated to a G4-binder, are quite rare. In fact, the first proof-of-concept for two novel G4-binders [^64^Cu]NOTA-TP, with chemo-radio-theranostic properties, was reported in our previous *in vitro* studies (15, 16).

To better evaluate the antitumor activity and toxicity of this new class of radiotheranostic agent, *in vivo* studies were performed with [^64^Cu]NOTA-C3-TP. Preclinical imaging by PET of [^64^Cu]NOTA-C3-TP provides good quality tumor contrast static images at 4, 24, and 48 h, that allow the tracer distribution to be visualized and quantified in nude mice bearing P53 (+/+) HCT116 tumors (Figure 5). The image quality of [^64^Cu]NOTA-C3-TP is due to, (1) its good tumoral uptake, (2) a long residency time in tumor, and (3) rapid elimination of the tracer from blood circulation and other normal surrounding tissues, which reduces potential background signal. The critical organ for early elimination of [^64^Cu]NOTA-C3- TP, was the kidney, with a maximum uptake of 22.6 %ID/g at ~5 min post-injection (Figure 3A, green line).

The biological half-life of [^64^Cu]NOTA-C3-TP in kidney was only ~2.5 h (Supplementary Figure S7). This high speed elimination from the kidney of positively charged [^64^Cu]NOTA-C3-TP is likely related to the small length of [^64^Cu]NOTA-C3-TP, that were measured as ~1.6 nm with Chimera software 1.15 (30). It is thus indicate that [^64^Cu]NOTA-C3-TP is small enough to pass through the glomerular basement membrane with the cut-off diameter of ~10 nm (31). After the 1st h post-injection, the critical organ for elimination of [^64^Cu]NOTA-C3-TP gradually becomes the liver as seen in static PET images at 4, 24, and 48 h (Figure 6). The biliary excretion of [^64^Cu]NOTA-C3-TP could perhaps derive from an enlargement of the complex due to binding with plasma proteins that cause it to exceed the renal cut-off limit. Our *ex vivo* results show that at 24 h, 96% of [^64^Cu]NOTA-C3-TP is bound to plasma protein (Supplementary Table S2). Since serum albumin has a total negative charge (−19) in physiological pH (7.4), that can be repelled by negatively charged of podocytes in the glomerular system and further prevent the filtration of [^64^Cu]NOTA-C3-TP bound to albumin (32).

The long lasting of [^64^Cu]NOTA-C3-TP in tumor (*t*_1/2_ > 48 h) (as opposed to normal tissues) is a substantial advantage not just for imaging but also for radiation dosimetry/therapy. There was only a 39% reduction in tumor uptake from the maximum value measured at 11.2 min post-administration (3.0 ± 0.2%) to the minimum value seen at 48 h (1.83 ± 0.40%). From a radiation dosimetry perspective, 48 h is the most critical time point, as almost 4-radioactive half-live of ^64^Cu have by then passed (33).

Interestingly, we noted a significant increase (~1.4-fold) in tumor uptake at all time points post-administration when a 100-fold excess of [^Nat^Cu]NOTA-C3-TP was co-injected with [^64^Cu]NOTA-C3-TP (Figure 7). Based on our observation that most [^64^Cu]NOTA-C3-TP becomes bound to plasma protein, we intuited that the simultaneous *i.v*. administration of [^Nat^Cu]NOTA-C3-TP with [^64^Cu]NOTA-C3-TP could release the radioactive platinum conjugate from plasma protein and result in an enhancement of [^64^Cu]NOTA-C3-TP tumor accumulation. This type of drug competition for interaction with plasma proteins including serum albumin is reported for various chemotherapeutic agents that binds to albumin *via* π-π interactions (34). Accordingly, co-injection with an excess of non-radioactive platinum conjugate could be employed in future studies to enhance the tumoral uptake, absorbed dose, and potentially, efficiency of treatment if no adverse effect is observed.

Our CRT results indicate that [^64^Cu]NOTA-C3-TP (137 MBq) postpones tumor growth by 3.8 ± 0.5 and 4.3 ± 1.0 days relative to vehicle and [^Nat^Cu]NOTA-C3-TP treated groups at 5Td, respectively. The tumor growth retardation (TGR) induced by [^64^Cu]NOTA-C3-TP (137 MBq) increased to 6 ± 2.0 days at 7Td (the time required for the tumor volume to increase by 7-fold relative to the initial volume). This result is consistent with earlier of Merle et al., who reported a significant radiosensitizing effect by a terpyridine platinum-derived G4-ligand under external beam radiation, in one cancer cell line and cancer-cell-xenografted-mouse model (35). From a dosimetric perspective, this TGR was obtained with 4-fold lower injected activity of [^64^Cu]NOTA-TP than with another ^64^Cu-based theranostic agent, reported by Anderson et al. (555 MBq) (33). Interestingly, the dose enhancement from 70 to 137 MBq retards the initial tumor growth from days 1 to 3 (Figure 8A). This result indicates that the initial “shoulder” of the tumor growth curve is expandable as a function of dose. This treatment pattern for [^64^Cu]NOTA-C3-TP could be employed for future dose fractionation. More importantly, the median survival of the mice treated with buffer (vehicle) and [^Nat^Cu]NOTA-C3-TP were, respectively, 9.5 and 13 days. The corresponding values for the mice treated [^64^Cu]NOTA-C3-TP (70 MBq) and [^64^Cu]NOTA-C3-TP (137 MBq) were significantly increased to 15 and 17 days, respectively (Figure 8B).

Regarding treatment with the [^64^Cu]NOTA-C3-TP, EFs of 1.9 ± 0.5 and 1.7 ± 0.5 were measured when 137 MBq was administrated relative to vehicle and [^Nat^Cu]NOTA-C3-TP groups, respectively. However, the accumulation of [^64^Cu]NOTA-C3-TP in tumor was very low, i.e., only 3.6 × 10^−4^ μg/g tumor 48 h post-injection. The absorbed radiation doses were also small, i.e., 1.7 ± 0.3 Gy for 70 MBq injected and 3.4 ± 0.4 Gy when treating with 137 MBq of the [^64^Cu]NOTA-C3-TP.

Our results were compared to treatments made with oxaliplatin alone and in combination with radiation therapy (18). Comparing the TGD in the same mouse model, for treatment with oxaliplatin and a radiation dose of 15 Gy (by external beam), with that for oxaliplatin alone, an EF of 5.18 was obtained. Tumors were irradiated at 48 h post-injection when the oxaliplatin in the tumor had reached a concentration of 0.12 μg/g of tumor (18). At the time these tumors were irradiated, the oxaliplatin in the tumor had reached 0.12 μg/g of tumor (18). While this corresponds to 333 times more platinum drug in the tumor with oxaliplatin than with [^64^Cu]NOTA-C3-TP; a much lower radiation dose (by a factor 4.4–8.8) is delivered when oxaliplatin is combined with a 15 Gy dose from an external beam (18). These results suggest that [^64^Cu]NOTA-C3-TP produces a better tumor response per amount of platinum drug accumulated in the tumor than is obtained with oxaliplatin in combination with radiation therapy.

The tumor uptake of [^64^Cu]NOTA-C3-TP was then compared to that of ^64^Cu(OAc)_2_ reported by Hueting et al. (36). The tumor uptake reported for ^64^Cu(OAc)_2_ increased from 1.28 ± 0.13 to 2.76 ± 0.63 %ID/g, at 15 min and 2 h post-injection, respectively, and reduced to 1.28 ± 0.07 %ID/g at 16 h (36). The tumor uptake obtained for [^64^Cu]NOTA-C3-TP is 2.3-fold higher than that of ^64^Cu(OAc)_2_ at earlier times, when most energy is deposited. Additionally, [^64^Cu]NOTA-C3-TP exhibits a higher retention in the tumor at times up to 48 h. From our previous work, ^64^Cu(OAc)_2_ showed significantly lower nuclear accumulation in HCT116 cells relative to ^64^Cu-NOTA-C3-TP (16). Together, the therapeutic effect of ^64^Cu-NOTA-TP is expected to be much higher than that of ^64^Cu(OAc)_2_ under similar conditions (activity, concentration and time window).

The amplification of the tumor response obtained with [^64^Cu]NOTA-C3-TP complex could be explained by the proximity between the radiation source, ^64^Cu, and the Pt drug, which promotes the production of LEEs by the Pt when exposed to the electrons generated by the ^64^Cu. As reported in the literature, these LEEs locally increase the density of DNA strand breaks, which would decrease the efficiency of their repair and thus amplify the cytotoxic efficiency per Pt atom accumulated in the DNA (37). In addition, [^64^Cu]NOTA-C3-TP is expected to bind to the G-quadruplex, which are highly vulnerable domains of DNA (11–13).

The pharmacokinetics of [^64^Cu]NOTA-C3-TP also differs from that of oxaliplatin. In our previous study (18), 10 mg/kg of oxaliplatin was injected per mouse, which corresponded to 25 nmol/g mouse. The quantity measured in the tumor at 48 h post-injection was 0.3 nmol/g, or 1.2% of the injected dose. When 0.02 nmol/g mouse of [^64^Cu]NOTA-C3-TP, was injected, the quantity accumulated in the tumor reached 4.4 × 10^−4^ nmol/g, corresponding to 1.8% of the injected dose. This suggests that [^64^Cu]NOTA-C3-TP shows a better capacity to accumulate in the tumor than does oxaliplatin. On the other hand, the injected dose of oxaliplatin was 1,250 times higher than that of [^64^Cu]NOTA-C3-TP, but its plasma concentration at 48 h post-injection was only about 6 times higher. These results indicate that [^64^Cu]NOTA-C3-TP clears more slowly than oxaliplatin does from mice which may favor its accumulation in the tumor. Moreover, the selectivity toward tumor (or therapeutic window) of [^64^Cu]NOTA-TP was better than that measured with oxaliplatin (Table 1).

The estimated absorbed radiation doses to the kidney and liver were, respectively, 6.0 ± 0.2 and 5.12 ± 0.06 Gy with [^64^Cu]NOTA-C3-TP, which are below the maximum tolerated dose (38). However, the radiation doses absorbed in these organs were 1.5–3.5 times higher than that in tumor, which reached 1.7 and 3.4 Gy, where they induced a partial tumor response, as expected. Further studies are therefore necessary to improve tumor accumulation for this new class of chemo-radio-theranostic agent, to induce a complete tumor response and acceptable absorbed doses to the liver and kidneys. In this case, the greatest advantage of [^64^Cu]NOTA-C3-TP would be for diagnosis and treatment of unresectable tumors or metastatic CRC.

By extrapolation of radiation dose per unit injected activity values (mGy/MBq) from mouse organs to a standard-sized adult human, we observed a considerable reduction in absorbed radiation in whole healthy organs (Table 2). This is consistent with the inverse proportionality between the mass of organ and measured S-value, that ultimately translates into absorbed dose (mGy) in standardized dosimetry calculations of OLINDA (25).

With respect to the high image contrast provided by [^64^Cu]NOTA-C3-TP, it can be concluded that this radio-theranostic agent has great potential as a novel, imaging tracer for future preclinical studies. More importantly, [^64^Cu]NOTA-C3-TP can greatly increase the anti-tumor response per amount of platinum drug accumulated in the tumor. To improve the therapeutic efficiency of this new class of radio-theranostic agent, its selectivity toward tumor and its tumor uptake must be enhanced. The conjugation of [^64^Cu]NOTA-C3-TP with a ligand that targets overexpressed receptors on colorectal cancer cells could be considered.

## Materials and methods

### Synthesis of [^64^Cu]NOTA-TP

Synthesis of NOTA-C3-TP, [^Nat^Cu]NOTA-C3-TP and [^64^Cu]NOTA-C3-TP were previously described by our group (14). Purity of the conjugates was verified by High Performance Liquid Chromatography (HPLC). All final compounds had an HPLC purity of ≥95% (Supplementary Figures S1A,B). Analytical HPLC were performed on an Agilent 1200 system (Agilent Technologies, Mississauga, Ontario, L5N 5M4, Canada) equipped with a Zorbax Eclipse XDB C18 reversed-phase column (4.6 × 250 mm, 5 μl) and an Agilent 1200 series diode array UV-Vis detector (Agilent Technologies) using method: flow = 1 mL/min; 0–23 min; 0–76.6% acetonitrile −0.025% TFA in H_2_O-0.05% TFA, 23–24 min; 100% acetonitrile, 24–30 min; 100–0% acetonitrile in H_2_O.

### [^64^Cu]NOTA-C3-TP

The preparation of [^64^Cu]Cu-NOTA-C3-TP was by incubating 250 μM of NOTA-C3-TP dissolved in 10% DMSO and 0.9% saline with 350–450 MBq (~10 nM) of ^64^Cu(OAc)_2_ in a total volume of 1–1.5 mL of 0.1 M ammonium acetate buffer, at pH 7.25, for ~20 min at room temperature. The radio chemical yield (>99%) was assessed by radio-TLC -and eluted on C18 plates using sodium citrate 0.1 M, pH = 5.5 (Supplementary Figure S1C).

### *In vivo* stability study

*In vivo* plasma stability and protein binding of [^64^Cu]NOTA-C3-TP were evaluated at 48 h after *i.v*. injection of 5–20 MBq in mouse by radio-TLC (39). Briefly, whole blood samples (1 mL) were collected from mice femoral artery *via* heparinized syringes tubes under deep anesthesia ~3% isoflurane in an air/oxygen mixture and then transferred to heparinized tubes. After 5 min centrifugation of the tubes containing whole blood ( × 2,000 g), plasma was gently isolated from blood cells for assessing the radio-TLC without further protein precipitation. The whole plasma was spotted on radio-TLC strips equipped with C18 plates. Free ^64^Cu(OAc)_2_ and [^64^Cu]NOTA-C3-TP were selected as controls in this experiment. According to the retention time (migration distance) related to each of these two controls, the percentage of ^64^Cu that released from [^64^Cu]NOTA-C3-TP was calculated. The radio-TLCs were eluted with 0.1 M sodium citrate buffer at pH 5.5 using an Instant Imager system (Bioscan, DC, U.S.A.) for the radiodetection.

### *Ex vivo* plasma protein binding study

*Ex vivo* plasma protein binding was investigated following 500 MBq incubation of [^64^Cu]NOTA-C3-TP compound in 0.5 mL of mouse plasma and 0.5 ml of PBS at 1, 4, 24, and 48 h post-incubation. Radioactive plasma was recovered from size exclusion tubes ranging from 10 to 50 KD. After 15 min centrifugation, radioactive plasma from each proteins fraction was gently transferred into a separate tube. Then, the porous membranes were washed with PBS three times to make sure that the proteins have been completely recovered. The absorbance spectrum (250–500 nm) of each protein fraction was carried out with a plate reader and compared to that of pure plasma. Radio-TLC analysis of the supernatant and the protein layers were carried out to confirm the results by spectrophotometry.

The binding of [^64^Cu]NOTA-C3-TP to plasma protein was assessed at 2, 4, 6, 16, 24, and 48 h by addition of pure acetonitrile (1/1) into the incubated [^64^Cu]NOTA-C3-TP to precipitate the total proteins. After 15 min centrifugation of the samples at 7,000 rpm, the supernatant was collected. The procedure was repeated twice to ensure the total precipitation of the proteins. The remained radioactivity in the supernatant as well as the precipitated proteins were measured in a dose calibrator and the protein binding percentage were calculated.

### Cell culture

The p53 wild-type HCT116 human colorectal cancer cells were bought from ATCC and cultured in Eagle's minimal essential medium (EMEM, Sigma-Aldrich, Oakville, Canada) supplemented with 10% fetal bovine serum, 1 mM sodium pyruvate and 2 mM L-glutamine in a humidified incubator of 5% CO_2_ at 37°C.

### Animal studies

All animal studies were approved by the Institutional and Animal Care and Use Committee of l'Université de Sherbrooke (N/Ref.2020-2611).

### Biodistribution in mice

Biodistribution of [^64^Cu]NOTA-TP was carried out in male Balb/c mice (19–23 g). 10–20 MBq of [^64^Cu]NOTA-C3-TP in 100–200 μL were administered intravenously (*i.v*.) in the caudal vein of the mice under isoflurane anesthesia. At 4, 24, and 48 h post-injection, the mice were euthanized under deep with ~3% isoflurane anesthesia in an air/oxygen mixture, followed by exposure to 5% CO_2_. At each time point, organs were collected, rinsed in 0.9% saline and the accumulated activity was counted by a Hidex automated gamma counter. The results for each organ were expressed as the average percentage of injected dose per gram of tissue (%ID/g) ± SD.

### PET imaging

For PET image acquisitions, a previous standard procedure using an animal PET-CT Triumph scanner (Gamma Medica Inc., Northridge, CA, USA) on HCT116 tumor-bearing nude (nu/nu) mice was followed (40). Before imaging, the mice were anesthetized with 1.5–2.0% isoflurane in an air/oxygen mixture and during the scan procedure isoflurane was maintained stable. Thirty seconds after starting the PET image acquisition, between 30 and 40 MBq (~17.5 nmol/kg) in 200 μL of the [^64^Cu]NOTA-C3-TP was injected *i.v*. in the caudal vein and a dynamic scan was performed for 1 h. The time duration of static scans were 20, 30, and 45 min at 4, 24, and 48 h post-administration of [^64^Cu]NOTA-C3-TP, respectively. The respiration rate and body temperature were monitored during each scan. Dynamic image reconstruction was carried out by using the maximum likelihood expectation maximization (MLEM-3D) procedure with 20 iterations [field of view (FOV) = 60 mm], with one background frame of 30 s followed by 19 × 180-s frames. For quantitative and qualitative analysis of PET images, the AMIDE software was employed (41). Accordingly, regions of interest (ROI) were traced over tumor, liver, kidneys, and muscle, then implemented on every frame of the dynamic image to obtain time activity curve (TAC). The percentage of injected dose per gram (%ID/g) of tissues were derived from the average ROI values detected by either 3-D iso-contour and circle modes of ROIs (3 × FWHM). The static image reconstruction was also carried out by using the MLEM procedure with 20 iterations and a single 20, 30, and 45-min frames at 4, 24, and 48 h, respectively.

### Tumor-to-organ ratio calculation

The ratios [(%ID/g)_tumor_/(%ID/g)_organ_] for tumor, liver, kidneys, and muscle, were calculated by ROI analysis of PET images.

### Scanner calibration

A cylindric phantom filled with 25 mL water (similar to the size of a mice) containing a known amount of ^64^Cu (~5 MBq) was used to calculate the calibration factor needed for the conversion of counts *per seconds* (CPS) into MBq per mL, from which the injected dose per gram of tissue (%ID/g) values were calculated. Accordingly, the decay corrected time-activity curves were plotted between the %ID/g and time (h).

### Chemo-radiation therapy

The CRT efficacy of [^64^Cu]NOTA-C3-TP compound was determined in male nude mice (19–23 g) inoculated subcutaneously (*s.c*.) with 1 × 10^6^ HCT116 cells. The animals were kept in animal facility under sterilized and pathogen-free conditions. When the tumor reached a diameter of 3–4 mm, mice were randomized and divided into four groups (3–5 animals per group). Animals in group 1 were considered as the control (injected with the vehicle), group 2 was injected with ^Nat^Cu-NOTA-C3-TP, groups 3 and 4 received [^64^Cu]NOTA-C3-TP at 70 MBq (1.89 mCi) and 137 MBq (3.78 mCi), respectively (equivalent of 17.5 nmol/kg). The groups 2, 3, and 4 received similar quantity of the drug (17.5 nmol/kg). Tumor volumes were calculated with the standard formula (42) as follows: tumor volume (mm^3^) = (L × W^2^)/2, where L is the longest and W the shortest diameter of the tumor in millimeters (mm), respectively. A diameter of 1.5 cm, an ulcerated necrotic center formed in the tumor, and any limitation on shoulder motion were considered as the limit for this study. The difference between antitumor activity of each complex was analyzed by independent (unpaired) sample *t*-test after plotting the relative tumor growth (%V_t_/V_0_) as a function of time (days). A relative body weight reduction (%W_t_/W_0_) higher than 20% was considered as a sign of toxicity (43). Kaplan-Meier survival curve was plotted between the start of treatment and the time that the mice were euthanized. The log-rank (Mantel-cox) test was used to compare the groups.

### *In vivo* radiation dosimetry

The effective and absorbed dose in critical organs associated with the elimination of [^64^Cu]NOTA-C3-TP compound as well as in the tumor were calculated by determining the area under the curve (AUC) up to 48 h after drug injection from graphs plotting the decay applied injected dose (MBq) as a function time (h) (formerly known as residence time). To extrapolate the AUC from 48 h up to infinity the following formula which represents the relationship between cumulated activity, Ã (MBq-hr), and the initial activity of a collection of radioisotopes, A_0_, was employed:

(1) $$\begin{array}{c} A\left(MBq-h\right)=1.44\times A_{0}\left(MBq\right)\times T_{1/2} \end{array}$$

(44)

The calculated AUC values were added from 48 h up to infinity, and then the outputs (in MBq/MBq × h) were calculated with the OLINDA software (OLINDA/EXM 1.1 software (Organ Level Internal Dose Assessment Code, Vanderbilt University, Nashville, USA) (25) to obtain the absorbed (mGy/MBq) and effective dose (mSv/MBq). This value was multiplied by the initial injected dose to calculate final absorbed (mGy) and effective dose (mSv).

In order to scale up the measured absorbed dose in mice to human, a standard extrapolation methodology described by Sparks et al. was followed (45).


$$\begin{array}{c} AUC_{human}{=AUC}_{mouse}\times \left[\frac{W_{human\;tissue}}{W_{human}}\times W_{mouse}\right] \end{array}$$

### Statistical analyses

Statistical analyses for the pharmacokinetics, tissue uptakes, tumor growth and CRT efficiency were performed using Excel software. Two-sided significance levels were calculated, and differences at *p* < 0.05 were considered statistically significant. The AUC were calculated using Graph pad prism [9.1.(221)]. All values are reported as mean ± SD.

## Data availability statement

The original contributions presented in the study are included in the article/Supplementary material, further inquiries can be directed to the corresponding author.

## Ethics statement

The animal study was reviewed and approved by Institutional and Animal Care and Use Committee of l'Université de Sherbrooke (N/Ref.2020-2611).

## Author contributions

Conceptualization: BG, MK, and LS. Methodology and investigation: MK and SA-M. Validation: BG, SA-M, and MK. Formal analysis, writing, and original draft preparation: MK and BG. Writing, review, and editing: MK, BG, BP, and LS. Supervision and funding acquisition: BG and LS. All authors have read and agreed to the published version of the manuscript.

## Funding

This work was financially supported by the Natural Sciences and Engineering Research Council of Canada (NSERC, RGPIN-2014-04354, and RGPIN-2019-05284) and the Canadian Institutes of Health Research (PJT-162325). MK received a scholarship from the Faculty of Medicine and Health Sciences, Université de Sherbrooke. BG is a member of the CRCHUS funded by the Fonds de recherche du Québec—Santé (FRQS) and holder of the Jeanne and J.-Louis Lévesque Chair in Radiobiology at Université de Sherbrooke.

## Conflict of interest

The authors declare that the research was conducted in the absence of any commercial or financial relationships that could be construed as a potential conflict of interest.

## Publisher's note

All claims expressed in this article are solely those of the authors and do not necessarily represent those of their affiliated organizations, or those of the publisher, the editors and the reviewers. Any product that may be evaluated in this article, or claim that may be made by its manufacturer, is not guaranteed or endorsed by the publisher.
