# Supplementary material for: In vivo behavior of [64Cu]NOTA-terpyridine platinum, a novel chemo-radio-theranostic agent for imaging, and therapy of colorectal cancer
Source: Front Med (Lausanne). 2022 Sep 23;9:975213. doi: 10.3389/fmed.2022.975213 (PMC9549809; doi:10.3389/fmed.2022.975213)
Supplement: Supplementary file 1 [file Data_Sheet_1.docx]

Supplementary Material

# Supplementary Figures and Tables

**
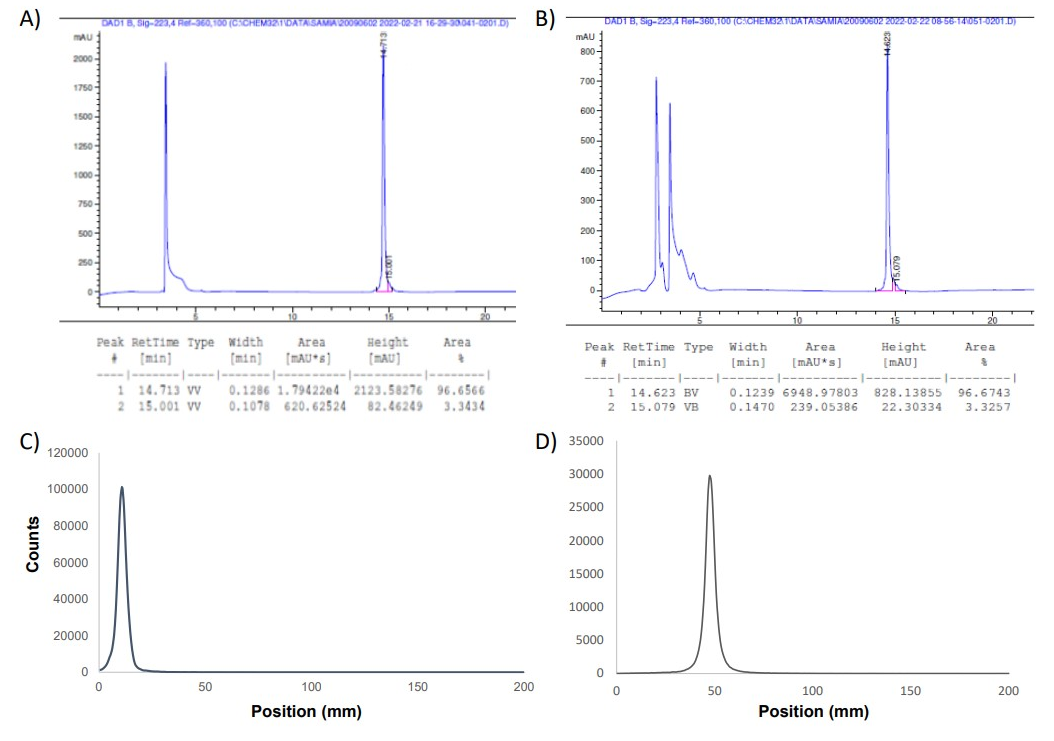
**

**Figure S1.** HPLC traces or HRMS spectra of NOTA-C3-TP (A) and [^Nat^Cu]NOTA-C3-TP (B);

**Figure S2.** Radio-TLC profiles of [^64^Cu]NOTA-C3-TP after 48h incubation in the whole plasma (A) and in the supernatant after protein precipitation (B).

**Figure S3.** Absorbance spectra of plasma before and after incubation with [^64^Cu]NOTA-C3-TP. (A) Absorbance spectrum of whole plasma (green line) and [^64^Cu]NOTA-C3-TP (red line) alone. (B) Supernatant containing 50-70 KD proteins (green line), those ranging between 10-50 KD (insert, blue line), and smaller than 10 KD (insert, orange line) after 24 h incubation of [^64^Cu]NOTA-C3-TP in mice.

**Figure S4.** Coronal PET images of [^64^Cu]-NOTA-C3-TP injected at 30 MBq, at 4 (A), 24 (B) and 48 h (C) p.i. in HCT116 human colorectal cancer bearing mice. Arrows point to tumors.


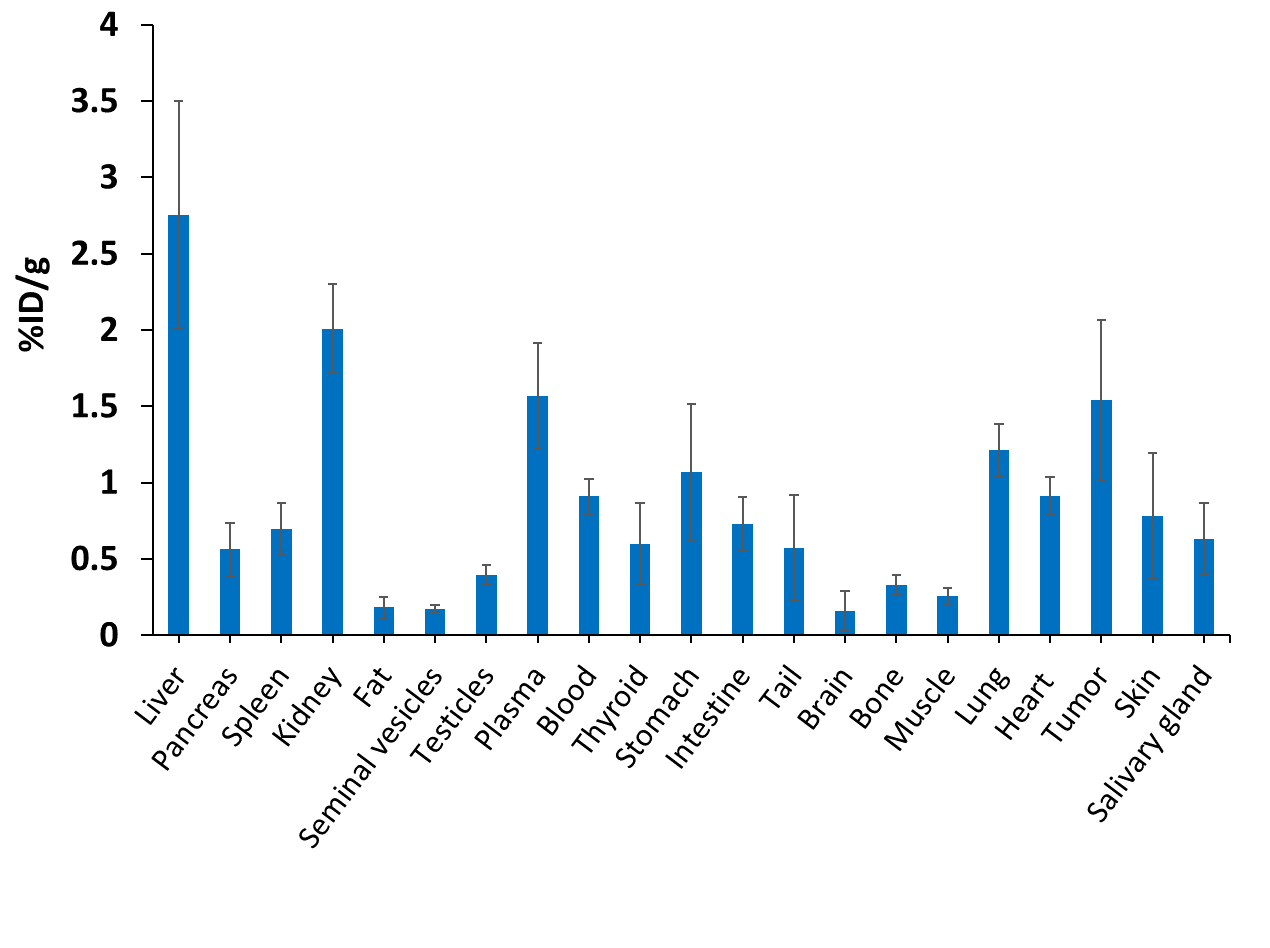


Figure S5. Biodistribution profile of [^64^Cu]NOTA-C3-TP after organ extraction in HCT116 tumor-bearing nude mice at 48 h post-injection.

**Figure S6.** Relative weight loss (%W_t_/W_0_) after treatment with 70 MBq (blue line) and 137 MBq (green line) of [^64^Cu]NOTA-C3-TP compared to [^Nat^Cu]NOTA-C3-TP (orange line) and the vehicle (yellow line).

**Slope (K) = -0.195**

**t_1/2_**

**Figure S7.** Elimination rate constant (K) and measured biological half-life of [^64^Cu]NOTA-C3-TP in kidney.

Table S1: In vivo PET-derived tumor to tissue ratios of [^64^Cu]NOTA-C3-TP in HCT116 tumor-bearing nude mice (n=7).

| Ratios | **Time after injection (h)** | | | |
| --- | --- | --- | --- | --- |
|  | **1** | **4** | **24** | **48** |
| Tumor/Liver | 0.63±0.13 | 0.55±0.11 | 0.76±0.22 | 0.91±0.33 |
| Tumor/Kidney | 0.28±0.05 | 0.68±0.15 | 1.20±0.19 | 1.20±0.34 |
| Tumor/Muscle | 5.05±1.5 | 6.80±1.80 | 15.20±4.60 | 12.30±4.90 |

**Table S2.** Percentage of [^64^Cu]-NOTA-C3-TP bound to plasma protein following incubation in mouse plasma (*ex-vivo*).

| **Incubation time (h)** | **Plasma binding**  **(%)** |
| --- | --- |
| 2 | 88 |
| 4 | 87 |
| 6 | 89 |
| 16 | 92 |
| 24 | 96 |
| 48 | 96 |
